# Generating 3D images of material microstructures from a single 2D image: a denoising diffusion approach

**DOI:** 10.1038/s41598-024-56910-9

**Published:** 2024-03-18

**Authors:** Johan Phan, Muhammad Sarmad, Leonardo Ruspini, Gabriel Kiss, Frank Lindseth

**Affiliations:** 1 Department of Computer Science, Norwegian University of Science and Technology, Trondheim, Norway; 2 Petricore Norway, Trondheim, Norway

**Keywords:** 3D image generation, Material microstructures, Denoising diffusion probabilistic models, Single-image-based generation, Solid Earth sciences, Materials science, Computer science, Solid Earth sciences, Materials science, Computer science

## Abstract

Three-dimensional (3D) images provide a comprehensive view of material microstructures, enabling numerical simulations unachievable with two-dimensional (2D) imaging alone. However, obtaining these 3D images can be costly and constrained by resolution limitations. We introduce a novel method capable of generating large-scale 3D images of material microstructures, such as metal or rock, from a single 2D image. Our approach circumvents the need for 3D image data while offering a cost-effective, high-resolution alternative to existing imaging techniques. Our method combines a denoising diffusion probabilistic model with a generative adversarial network framework. To compensate for the lack of 3D training data, we implement chain sampling, a technique that utilizes the 3D intermediate outputs obtained by reversing the diffusion process. During the training phase, these intermediate outputs are guided by a 2D discriminator. This technique facilitates our method’s ability to gradually generate 3D images that accurately capture the geometric properties and statistical characteristics of the original 2D input. This study features a comparative analysis of the 3D images generated by our method, *SliceGAN* (the current state-of-the-art method), and actual 3D micro-CT images, spanning a diverse set of rock and metal types. The results shown an improvement of up to three times in the Frechet inception distance score, a typical metric for evaluating the performance of image generative models, and enhanced accuracy in derived properties compared to *SliceGAN*. The potential of our method to produce high-resolution and statistically representative 3D images paves the way for new applications in material characterization and analysis domains.

## Introduction

Three-dimensional (3D) volumetric images are a critical resource in various disciplines, including geophysics, petroleum, and materials science, due to their role in the numerical analysis and computational modeling of materials’ internal structures. These data, represented as a 3D grid of voxels (volumetric pixels), provide an intricate view of the internal structure of diverse materials, which is vital for deriving physical properties in different industries.

The acquisition of 3D images often poses significant challenges. Traditional image acquisition methods, such as computed tomography (CT) scanners, require substantial financial investment and skilled operators. Conventional techniques like micro-CT are often limited by the resolution capabilities of the imaging equipment. Practical issues related to sample preparation can further complicate acquiring high-resolution 3D images for specific materials or structures. Micro-CT scanners, which utilize X-rays, may also encounter difficulties penetrating radiodense materials, particularly metals, creating additional challenges in capturing comprehensive 3D images of such materials.

In contrast, sub-micrometer 3D scanning solutions such as nano-CT^1^ and FIB-SEM (Focused Ion Beam Scanning Electron Microscope)^2^ may offer higher resolution capabilities. However, these technologies come with a significantly higher price tag compared to micro-CT, have limited scanning sample sizes, and face limitations related to image quality, inhibiting their widespread application across various sectors within academia and industry^3^^4^.

Given these challenges, there has been growing interest in developing techniques that can generate 3D volumetric data using 2D images. This approach offers a promising alternative, as 2D imaging methods such as optical microscopes or Scanning Electron Microscopes (SEM) are in many cases more cost-effective and flexible in terms of resolution capabilities, including at the nanoscale^5^. Currently, 2D images are primarily used alongside 3D images for quality control or as supplementary material when the resolution of 3D imaging fails to fully capture the studied sample’s structure. Consequently, the capability to create 3D images from 2D images could reduce costs associated with the imaging process, enhance accessibility, and improve the efficiency of 3D image generation across diverse scientific fields.

**Figure 1:**
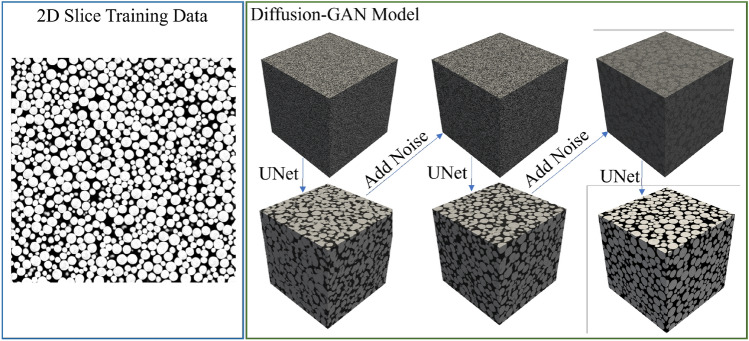
Diffusion-GAN model: the proposed method is based on a denoising diffusion process combined with a generative adversarial framework. In this setting, at test time, starting from a cube of noise, the noise is iteratively estimated using a Unet, removed and then added back to the sample. This process is repeated to obtain a noise-free representative sample. Our method can learn from only a few 2D slices of the training image, as shown on the left.

Most previous approaches for generating 3D voxelized data from 2D input require learning from 3D image data. Our work belongs to the category of methods that generate 3D images from a single 2D input, as shown in Fig. 1. In addition, they can do so by learning from 2D images. The uniqueness of our work is that we propose a framework that utilizes a denoising diffusion-based probabilistic model (DDPM)^6^. Since DDPM requires ground truth (GT) data for training, we propose modifications to enable it to operate without needing 3D GT. This is achieved through utilizing the reverse diffusion process (chain sampling). In addition, we utilize the generative adversarial network (GAN) loss^7^ since it helps to generate realistic samples. GANs can be unstable when used in a standalone setting to learn from 2D images generating 3D images. However, we propose to stabilize training by combining GANs with diffusion models.

Additionally, addressing the practical requirements of 3D image generation for industrial applications, we have adapted the diffusion process to converge into the final image with just a few denoising steps, specifically 11 in this study, as opposed to the thousands of steps typically used in 2D image generation. This modification is crucial for reducing computation time when working with 3D data, where a large image with over a billion voxels ($1000 \times 1000 \times 1000$) is often necessary for comprehensive material characterization.

Our results prove to be more accurate than previous works in both visual quality and physical/statistical properties. We further showed that our model can successfully learn to generate 3D images from a single 2D input across a wide variety of cases, ranging from rocks to metal alloys.

The contributions of our work can be summarized as follows:

- We propose a method based on the Denoising Diffusion Probabilistic Model (DDPM) for generating 3D microstructures from a single 2D image.
- We demonstrated the feasibility of applying DDPM without the need for training on GT data.
- Our method significantly outperforms existing approaches in terms of visual quality and statistical properties. Moreover, it demonstrates robust performance even on complex images with high heterogeneity, where current state-of-the-art methods fail.

The ability to generate 3D images from a single 2D image would allow us to perform characterizations and analyses that require the availability of 3D data. This technique would be suitable for application in cases where micro-CT imaging is not feasible, such as when capturing features at sub-micrometer resolution or for materials without any density contrast, or in the case of high-density materials like metals^5^.

### Related works

Creating 3D models or images of specific porous structures or materials has been a long-standing research challenge since the advent of image-based numerical analysis. Existing methodologies for tackling this problem can be broadly classified into three main categories: process-based modeling, properties-based generation, and machine learning-based generation.

#### Process-based modeling

Process-based modeling approaches aim to emulate the mechanisms underlying the natural formation of materials. In these models, the physical and chemical processes that occur during material formation, such as deposition, compaction, cementation, dissolution, and fracturing, are translated into mathematical and computational algorithms. By closely imitating these natural processes, process-based models allow for extensive control over the properties and characteristics of the generated samples, making them useful for hypothesis testing or simulating a wide array of possible scenarios^8–13^.

Despite their benefits, process-based models also have certain limitations. Simulating natural processes with satisfactory accuracy is computationally intensive, time-consuming, and challenging due to the complex interplay of numerous factors and the stochastic nature of many processes. Furthermore, operating these models requires a comprehensive understanding of the processes being replicated and the simplification of actual phenomena. As a result, there can be substantial discrepancies between the structures produced by these models and the real materials.

#### Properties-based generation

Generating 3D images can also be achieved through an iterative generation process that aims to converge toward a structure with desired statistical properties. This approach encompasses both stochastic-based modeling and optimization-based modeling^14–16^ where statistical descriptors such as the Minkowski functional and the n-point correlation function are commonly used. One of the main advantages of this method is its capability to generate models with specific desired properties. However, this approach is restricted to binary segmented images since most statistical descriptors for images are specifically designed for binary data. In more complex scenarios, especially for heterogeneous material, accurately capturing non-statistical representative features becomes challenging, potentially leading to the generation of unrealistic images even when the material’s statistical properties are matched.

#### Machine learning-based generation

The recent advancements in 3D image generation have predominantly focused on utilizing machine learning techniques with existing 3D data to generate new images. One prominent approach in this field is the use of Generative Adversarial Networks (GANs)^7^. GANs have been applied for unconditional generation^17,18^ and conditional generation^19–23^, ? of 3D images for micro-CT data. However, training a GAN-based model requires careful attention to ensure stability^24,25^. Challenges such as mode collapse and catastrophic forgetting can arise when using GANs for conditional generation tasks, necessitating the incorporation of additional consistency loss^26,27^.

To overcome the limitations of GAN-based methods, hybrid models that combine transformers and VQ-VAEs have emerged as an alternative solution. These models offer stable training and the ability to generate high-fidelity 3D rock samples from 2D conditional images^28^.

However, all of the mentioned works rely on the availability of 3D GT data for training, which can pose limitations in terms of accessibility, particularly when dealing with samples that contain a significant number of sub-micrometer features. In a recent study, Kench et al.^29^ showcased the capability of generating 3D microstructures with only 2D images as training data. Nevertheless, their approach relied on GANs, which are prone to common issues like unstable training and mode collapse.

In contrast to existing approaches, we propose a novel and stable diffusion-based method that achieves 3D image generation of material microstructures using only a single 2D image.

### Background

#### Denoising diffusion probabilistic models

This section provides a basic understanding of Denoising Diffusion Probabilistic Models (DDPM), also known as diffusion models, which serve as the foundation for our proposed method. DDPM consists of two main processes: the forward and reverse processes^6^.

In the forward process, noise, typically Gaussian noise, is gradually added to the data distribution $q({\textbf{x}}_0)$, where ${\textbf{x}}_0$ is the noise-free target. This process proceeds step by step, with the variance of the added noise changing according to a predefined schedule $\beta _t$ ($\beta _1, \ldots , \beta _T$). The forward process can be expressed as follows:

1 $$\begin{aligned}{}&q({\textbf{x}}_{1: T} | {\textbf{x}}_{0})=\prod _{t\ge 1} q({\textbf{x}}_{t} | {\textbf{x}}_{t-1})\\&\quad ={\mathcal {N}}({\textbf{x}}_{t}; \sqrt{1-\beta _{t}} {\textbf{x}}_{t-1}, \beta _{t} {\textbf{I}}), \end{aligned}$$

In the reverse process, the aim is to recover the data from noise in steps. A diffusion model is required, which is parameterized by $\theta$ with mean $\varvec{\mu }{\theta }({\textbf{x}}{t}, t)$ and variance $\sigma ^2_t$. The reverse denoising process is given as:

2 $$\begin{aligned} p_{\theta }({\textbf{x}}_{0: T})=p({\textbf{x}}_{T}) \prod _{t\ge 1} p_{\theta }({\textbf{x}}_{t-1} | {\textbf{x}}_{t})\\={\mathcal {N}}({\textbf{x}}_{t-1}; \varvec{\mu }_{\theta }({\textbf{x}}_{t}, t), \sigma ^2_t {\textbf{I}}), \end{aligned}$$

To train this model, the variational bound on the negative log-likelihood objective $p_{\theta }({\textbf{x}}_{0})$ is optimized, defined as $\int p_{\theta }({\textbf{x}}_{0: T}) d {\textbf{x}}_{1: T}$. The variational lower bound is equivalent to matching the true denoising distribution $q({\textbf{x}}_{t-1} | {\textbf{x}}_{t})$ with the parameterized denoising model $p_{\theta }({\textbf{x}}_{t-1} | {\textbf{x}}_{t})$ using the loss function:

3 $$\begin{aligned} {\mathcal {L}} = -\sum _{t \ge 1} {\mathbb {E}}_{q({\textbf{x}}_{t})}\left[ D_{\textrm{KL}}\left( q({\textbf{x}}_{t-1} | {\textbf{x}}_{t}) \Vert p_{\theta }({\textbf{x}}_{t-1} | {\textbf{x}}_{t})\right) \right] + C \end{aligned}$$

where $D_{\textrm{KL}}$ represents the Kullback-Leibler (KL) divergence between the two distributions, i.e., the true denoising distribution $q({\textbf{x}}_{t-1} | {\textbf{x}}_{t})$ and the parameterized denoising model $p_{\theta }({\textbf{x}}_{t-1} | {\textbf{x}}_{t})$. *C* is a constant.

Two fundamental assumptions are commonly made in diffusion models: First, the denoising distribution $p_{\theta }({\textbf{x}}_{t-1} | {\textbf{x}}_{t})$ is modeled as a Gaussian distribution. Second, the number of denoising steps *T* is assumed to be large.

#### Denoising diffusion GANs

To address the challenge of requiring a large number of denoising steps in diffusion models, Xiao et al.^30^ proposed a combination of Denoising Diffusion Probabilistic Models (DDPM) and Generative Adversarial Networks (GANs). Their work introduced two major modifications to the original diffusion process:

- Adversarial Loss: Instead of using typical loss functions like Mean Squared Error (MSE) or Mean Absolute Error (MAE), this method used an adversarial loss from a conditional discriminator.
- Direct Output of Noise-Free Images: Rather than training the DDPM to output the noise for a given image, which is then subtracted to obtain the noise-free image, this method directly generates the noise-free image.

By combining DDPM with GANs and implementing the mentioned modifications, the method proposed by Xiao et al. achieved a drastic reduction in the number of denoising steps required by a factor of $10^3$, while also improving the quality of the generated images and maintaining stable training.

## Methods

Denoising diffusion models alone are inherently unsuitable for solving the problem of generating 3D images from a single 2D image, as they require a noise-free GT image ${\textbf{x}}_0$ for training. The absence of ${\textbf{x}}0$ renders the forward process $q({\textbf{x}}{1:T}|{\textbf{x}}_0)$ mentioned in Eq. (1) invalid. Consequently, to adapt DDPM to our specific problem, we changed the original DDPM pipeline to cater to learning with only 2D GT data.

**Figure 2:**
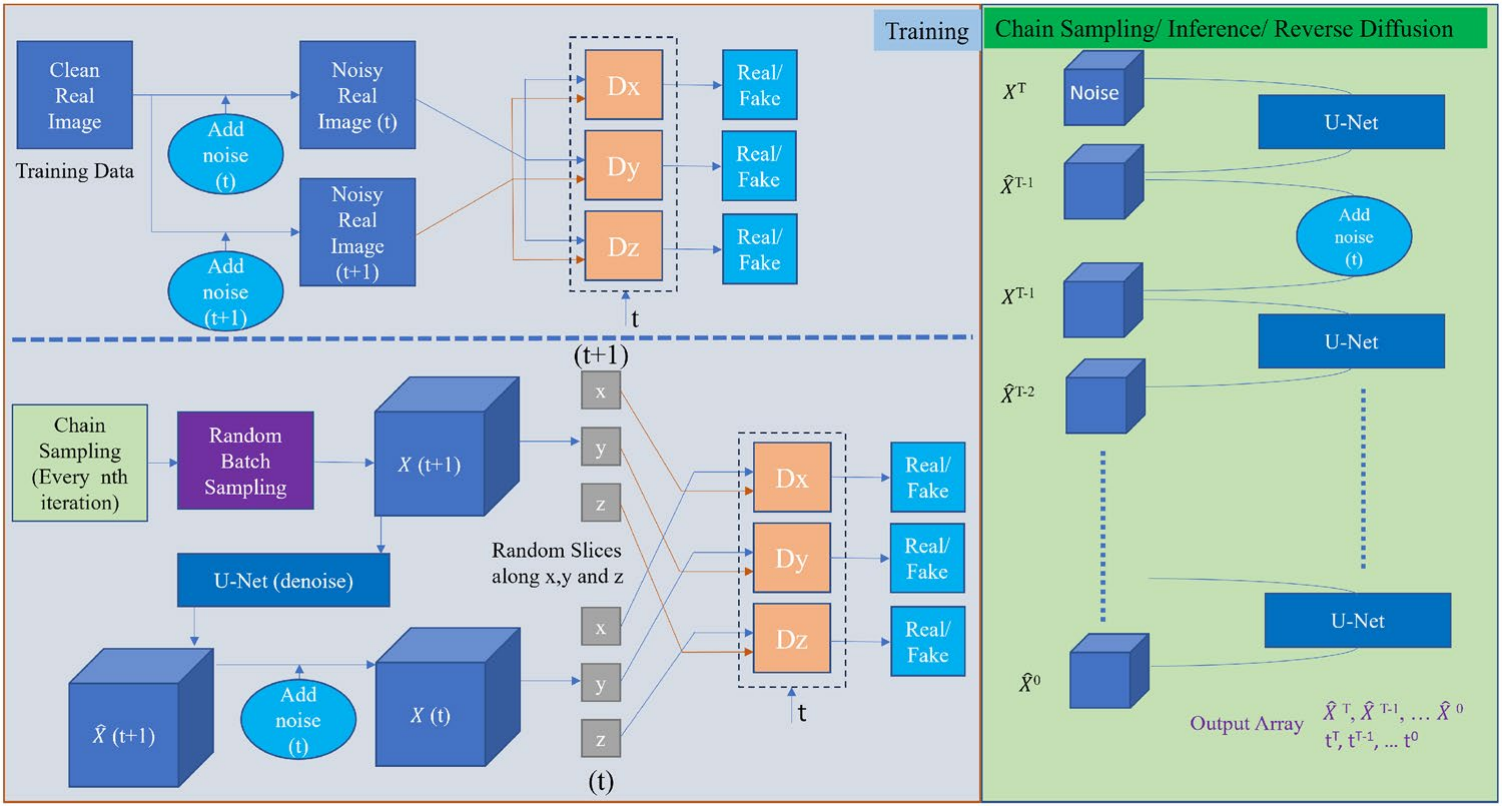
Our method: this figure shows the overview of our diffusion GAN based model for 3D generation using only 2D data for training.

Inspired by the *SliceGAN* architecture introduced by Kench et al.^29^, which utilized a 2D discriminator as the adversarial loss for a 3D generator, we have adapted the method proposed in the Denoising Diffusion GANs work by Xiao et al.^30^ to a similar setting as *SliceGAN*. An overview of our method is shown in Fig. 2. The main difference between SliceGAN and our method is that SliceGAN uses only generative adversarial networks. However, we use the diffusion model with GANs to get a more stable and robust training pipeline. However, using the diffusion model for this task is not trivial. Therefore, we provide a novel method to deploy diffusion models for 3D image generation from 2D slices.

### Chain sampling as a means of data generation

The lack of a noise-free GT ${\textbf{x}}_0$ presents a significant challenge when adapting the Denoising Diffusion Probabilistic Models (DDPM) framework to the task of generating 3D images from a single 2D input. To overcome this challenge, we propose a novel approach called chain sampling, which involves leveraging the reverse diffusion process during training alongside the forward diffusion process. This departure from the conventional usage of the reverse process solely for inference or testing purposes is a key distinction in our method. By employing chain sampling, we can utilize intermediate results during training as a substitute for the missing noise-free 3D GT ${\textbf{x}}_0$, under the assumption that the denoising model is still progressing in the correct direction. The chain sampling process, illustrated in Fig. 2, involves adding the corresponding level of noise using a simplified noise addition process as shown in Eq. (4). The denoising model G then performs the denoising operation on the image, as described in Eq. (5).

4 $$\begin{aligned} {\textbf{x}}_{t}&= q({\hat{{\textbf{x}}}}_{t+1}) = \beta _{t}*{\hat{{\textbf{x}}}}_{t+1} + (1-\beta _{t})*{\mathcal {N}}(\varvec{\mu }_{\theta }({\hat{{\textbf{x}}}}_{t+1}, t+1), \sigma ^2_t) \end{aligned}$$

5 $$\begin{aligned} {\hat{{\textbf{x}}}}_{t}&= {G}(x_{t}, t) \end{aligned}$$

#### Discriminator setting

To ensure the training of our denoising model in the absence of noise-free GT ${\textbf{x}}_0$, we employ a 2D discriminator trained on both the 2D image and the intermediate results from the chain sampling process. Similar to any GANs-based architecture, our discriminator requires both real data and fake (generated) data to train:

*Fake Data*: In the lower part of Fig. 2, we illustrate the process for generating fake data. During each training iteration, we sample an image from the output array of the chain sampling process, which corresponds to a denoised generated image ${\hat{{\textbf{x}}}}_t$. From this image, we randomly select a slice from each axis (*X*, *Y*, *Z*) and feed these slices to their respective discriminators $D_{x}, D_{y}, D_{z}$ (Eq. 6). While it is possible to use a single discriminator, we have found that utilizing three separate discriminators leads to more stable training and enables us to handle asymmetrical images effectively.

6 $$\begin{aligned} D_{\phi }({\textbf{x}}_{t-1}^{3D}, {\textbf{x}}_t^{3D}, t) =D_{x}({\textbf{x}}_{t-1}^{3Dx}, {\textbf{x}}_t^{3Dx}, t) \quad \\ +D_{y}({\textbf{x}}_{t-1}^{3Dy}, {\textbf{x}}_t^{3Dy}, t) + D_{z}({\textbf{x}}_{t-1}^{3Dz}, {\textbf{x}}_t^{3Dz}, t) \end{aligned}$$

*Real data*: In the upper part of Fig. 2, we depict the process of generating real data for training the discriminator. Since our discriminator consists of 2D convolutional layers, we can easily add different levels of noise into the 2D image to retrieve the corresponding ${\textbf{x}}_{t-1}$ and ${\textbf{x}}_t$. These noisy images, with noise added according to predefined $\beta _t$ values, serve as the real data inputs for training the discriminator.

7 $$\begin{aligned}{} & {} D_{\phi }({\textbf{x}}_{t-1}^{2D}, {\textbf{x}}_t^{2D}, t) = D_{x}({\textbf{x}}_{t-1}^{2D}, {\textbf{x}}_t^{2D}, t) \nonumber \\{} & {} \quad +D_{y}({\textbf{x}}_{t-1}^{2D}, {\textbf{x}}_t^{2D}, t) + D_{z}({\textbf{x}}_{t-1}^{2D}, {\textbf{x}}_t^{2D}, t) \end{aligned}$$

8 $$\begin{aligned}{} & {} \min _{\theta } \sum _{t \ge 1} {\mathbb {E}}_{q({\textbf{x}}_{t})}\left[ D_{\textrm{x}}\!\left( q({\textbf{x}}_{t-1}^{2D} | {\textbf{x}}_{t}^{2D}) \Vert q_({\textbf{x}}_{t-1}^{3Dx} | {\textbf{x}}_{t}^{3Dx})\right) \right. \nonumber \\{} & {} \quad \left. + D_{\textrm{y}}\!\left( q({\textbf{x}}_{t-1}^{2D} | {\textbf{x}}_{t}^{2D}) \Vert q_({\textbf{x}}_{t-1}^{3Dy} | {\textbf{x}}_{t}^{3Dy})\right) \right. \nonumber \\{} & {} \quad \left. + D_{\textrm{z}}\!\left( q({\textbf{x}}_{t-1}^{2D} | {\textbf{x}}_{t}^{2D}) \Vert q_({\textbf{x}}_{t-1}^{3Dz} | {\textbf{x}}_{t}^{3Dz})\right) \right. ], \end{aligned}$$

To manage the computational intensity of generating large 3D images, we had to restrict the number of denoising timesteps to a smaller value, specifically $T=11$. Consequently, this resulted in larger $\beta _t$ values for each diffusion step. Since our approach involved a significantly reduced number of denoising timesteps compared to the original Denoising Diffusion GANs, we paid close attention to selecting suitable $\beta _t$ values. The aim was to maintain a similar level of denoising complexity for each step, despite the reduced overall number of steps.

For the adversarial training, we define the sum of the three time-dependent discriminators as $D_{\phi }({\textbf{x}}_{t-1}, {\textbf{x}}_t, t): {\mathbb {R}}^N \times {\mathbb {R}}^N \times {\mathbb {R}} \rightarrow [0, 1]$, with parameters $\phi _x$, $\phi _y$, and $\phi _z$, as shown in Eq. (8). This discriminator takes the *N*-dimensional 2D slices ${\textbf{x}}_{t-1}^{2D}$ and ${\textbf{x}}_t^{2D}$ of ${\textbf{x}}_{t-1}$ and ${\textbf{x}}_t$ as inputs and determines whether the input is a plausible denoised version of ${\textbf{x}}_t^{2D}$ or not.

## Experiments

### Data

In this study, we used a combination of internal data and publicly available data to evaluate the performance of our model. In the first case, we validated the quality of the generated images compared to 3D GT (Table 1) using four micro-CT 3D images: a Glass Bead image, two sandstone images with different resolutions, and a Savoniere carbonate image from the digital rock portal^31^. For each 3D image, we randomly selected five unrelated 2D slices to train our model.

**Table 1:** Measured FID score across three dimensions (x, y, z) between the generated image and the GT, the closer to 0 the better.

Rock type	Dimension	Our (x, y ,z)			SliceGAN (x, y ,z)		
Glassbead	$200\times 200\times 200$	54.78	60.95	59.67	87.37	99.02	72.33
Bentheimer sandstone	$256\times 256\times 256$	35.62	49.13	40.99	46.91	61.59	54.65
Sandstone	$500\times 500\times 500$	23.58	23.81	20.46	25.25	29.82	21.76
Savoniere	$250\times 250\times 250$	171.57	186.60	172.20	476.33	436.95	435.58

In the second case (Table 2), we considered scenarios where only a single 2D image was available for both training and evaluation. The images used in this case include a Cast Iron with magnesium-induced spheroidized graphite and a Brass (Cu 70%, Zn 30%) with recrystallized annealing twins from Microlib^32^. Both images were captured using reflected light microscopy^33^. In addition, we also used an SEM image of kaolinite clay minerals.

**Table 2:** Measured FID score across three dimensions (x, y, z) between the generated image and the GT.

Material type	Dimension	Our (x, y ,z)			SliceGAN (x, y ,z)		
Magnesium treated cast iron	$800\times 528$	60.03	62.90	63.37	63.81	67.89	66.04
Brass (Cu 70%, Zn 30%)	$542\times 800$	107.18	97.37	109.18	266.78	252.17	216.54
Kaolinite clay mineral	$256\times 256$	177.78	177.21	183.78.65	360.52	335.13	317.98

### Evaluation metric

We utilize Fréchet Inception Distance (FID) as our evaluation metric^34^. FID is a popular choice for assessing the quality of generated images in tasks such as GAN evaluations. It can serve as a measure of similarity between two datasets of images. The FID metric calculates the Fréchet distance between two multivariate Gaussian distributions that are fitted to feature representations of the Inception network. One distribution represents real images, while the other represents generated images. The lower the FID score, the more similar the two datasets of images are in terms of their distribution in the high-dimensional space defined by the Inception network. Hence, a lower FID signifies a higher quality of generated images. It captures how well the generated images mimic the real ones.

### Resources and hyper-parameters

Each experiment in this study is conducted using PyTorch on a single Nvidia RTX 3090 GPU. The training time for both *SliceGAN* and our method was set to 24 hours. In our experiments, we used 11 denoising time steps ($T = 11$) with corresponding $\beta _t$ values ranging from 0.9100 to 0.0000, as follows: [0.9100, 0.8109, 0.7058, 0.5985, 0.4929, 0.3931, 0.3025, 0.2238, 0.1586, 0.1070, 0.0685, 0.0000].

## Results and discussions

### Glass beads generation

Our study introduces a novel 3D image generation method, the efficacy of which we assessed through a comparative analysis with glass bead pack images. These images, which naturally depict spherical formations in a densely packed array, are reduced to varying sizes of 2D circles in their planar representations. Our validation approach involved contrasting our method’s output with results from similar studies, focusing specifically on the fidelity of reconstructing 3D spherical shapes from these 2D circular projections. For this purpose, we selected benchmark studies by^22,29,35,36^ and^18^ for comparison.

**Figure 3:**
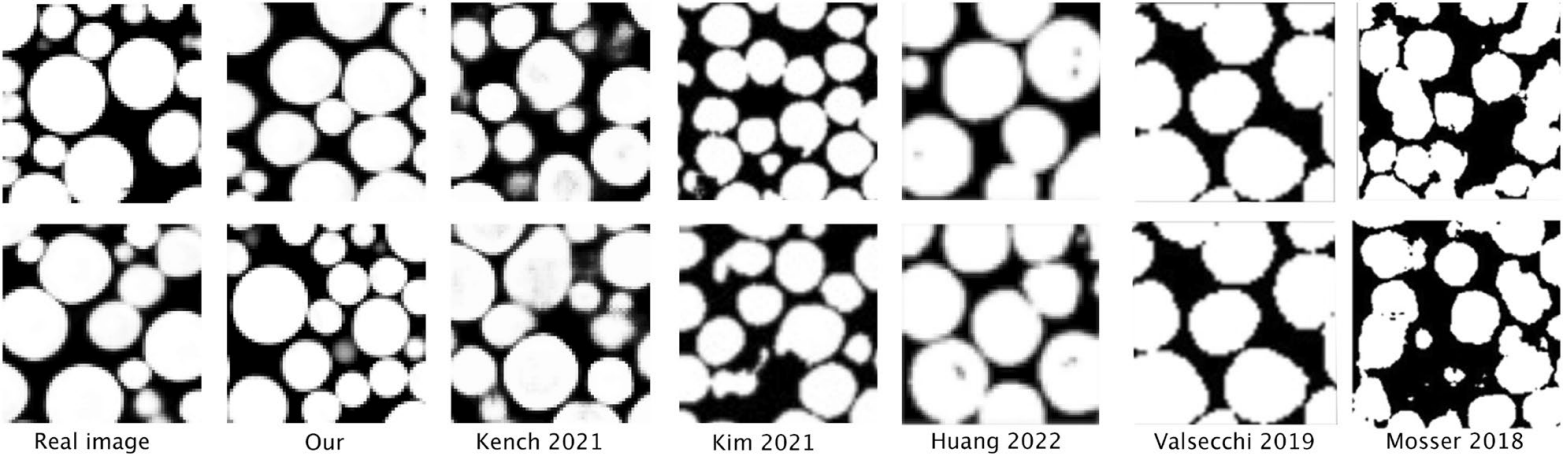
Visual comparison of spherical shape generation from 2D circular inputs in glass bead packs. This study contrasts our method’s results with previous deep learning-based 3D image generation techniques, highlighting our approach’s enhanced accuracy in generating spherical shapes.

Figure 3 showcases the cross-sectional views of structures synthesized using our method, alongside those generated by an authentic glass bead image, the SliceGAN algorithm, and the methods employed in the aforementioned studies. This highlights our method’s unique capability in accurately rendering spherical shapes in 3D from 2D inputs, a feature distinctively absent in the comparative methods, especially in terms of artifact-free shape generation.

**Figure 4:**
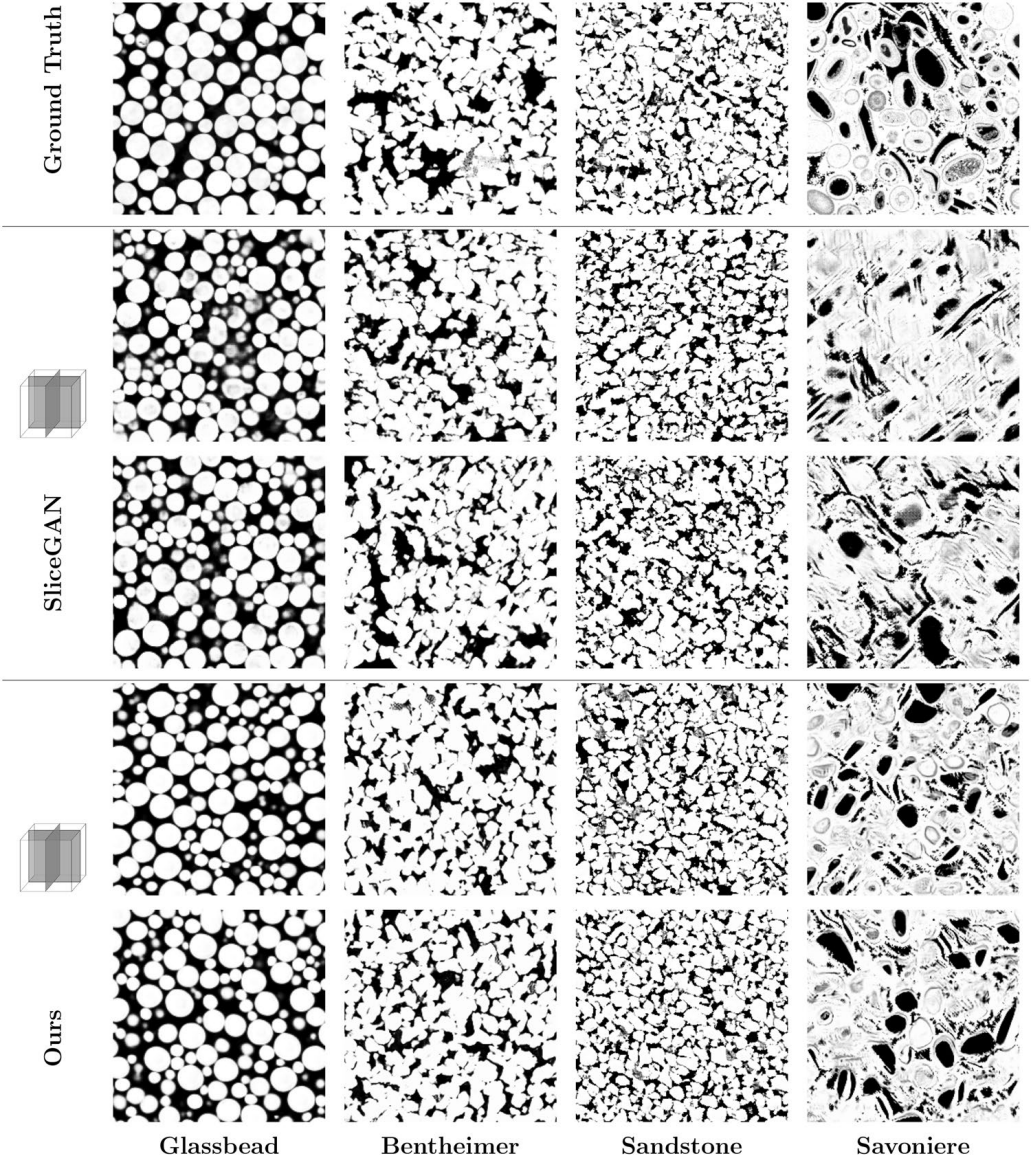
Visual comparison with micro-CT images: cross-sections of 3D images generated by our method and SliceGAN, alongside their respective ground truth or training data. The GT images are 3D X-ray microCT scans obtained at varying resolutions. The Glassbeads case showcases our method’s superior performance over SliceGAN. Our model can capture the spherical shape of the object, even though it only sees circles at the 2D input. In more challenging cases like the Savoniere—a carbonate of fossilized microorganism—our method proves its robustness by generating images that bear a higher resemblance to reality, despite the heterogeneous nature of the original image.

### Comparison with 3D GT

#### Visual comparison and FID score

Our first case study used four different 3D micro-CT images to evaluate both the visual quality and the accuracy of the characterized properties of our generated 3D images against the GT. For each image, five 2D slices of the xy plane, taken from different locations along the z-axis, were used to train our model and *SliceGAN*. We chose to use five 2D images since a single 2D slice might not fully capture the range of structural variation present in the 3D image. A step length of 11 was selected for our model to ensure fast generation times for large images. During each training iteration, we used a batch of random 64x64 pixel crops as input, which subsequently produced outputs of 64x64x64. The cross-sections of the images used in the training, as well as the images generated by both methods, are shown in Fig. 4.

In assessing the performance of our method compared to *SliceGAN*, we used the Frechet inception distance (FID) scores as a measure of visual quality^37^. To compute the FID score, the original requirement was for 2D images as input. To adapt this calculation to 3D images, we treated them as stacks of 2D images and computed the FID score across three dimensions (x, y, z). In comparison to other studies that used the FID score for image generation evaluation, the FID scores presented in Table 1 are notably higher. These higher FID scores are due to the few slices from the 3D image used for training not being able to cover the real data distribution of the 3D GT, especially for heterogeneous materials like the Savoniere Carbonate.

#### Comparison of porous media properties

In the context of porous media, it is crucial to evaluate our model’s performance in terms of physical properties. To calculate these properties, we used Porespy^38^, an open-source tool specifically designed to analyze 3D images of porous materials. With Porespy, we calculated local porosity, the two-point correlation function, and pore size distribution of the images depicted in Fig. 4.

**Figure 5:**
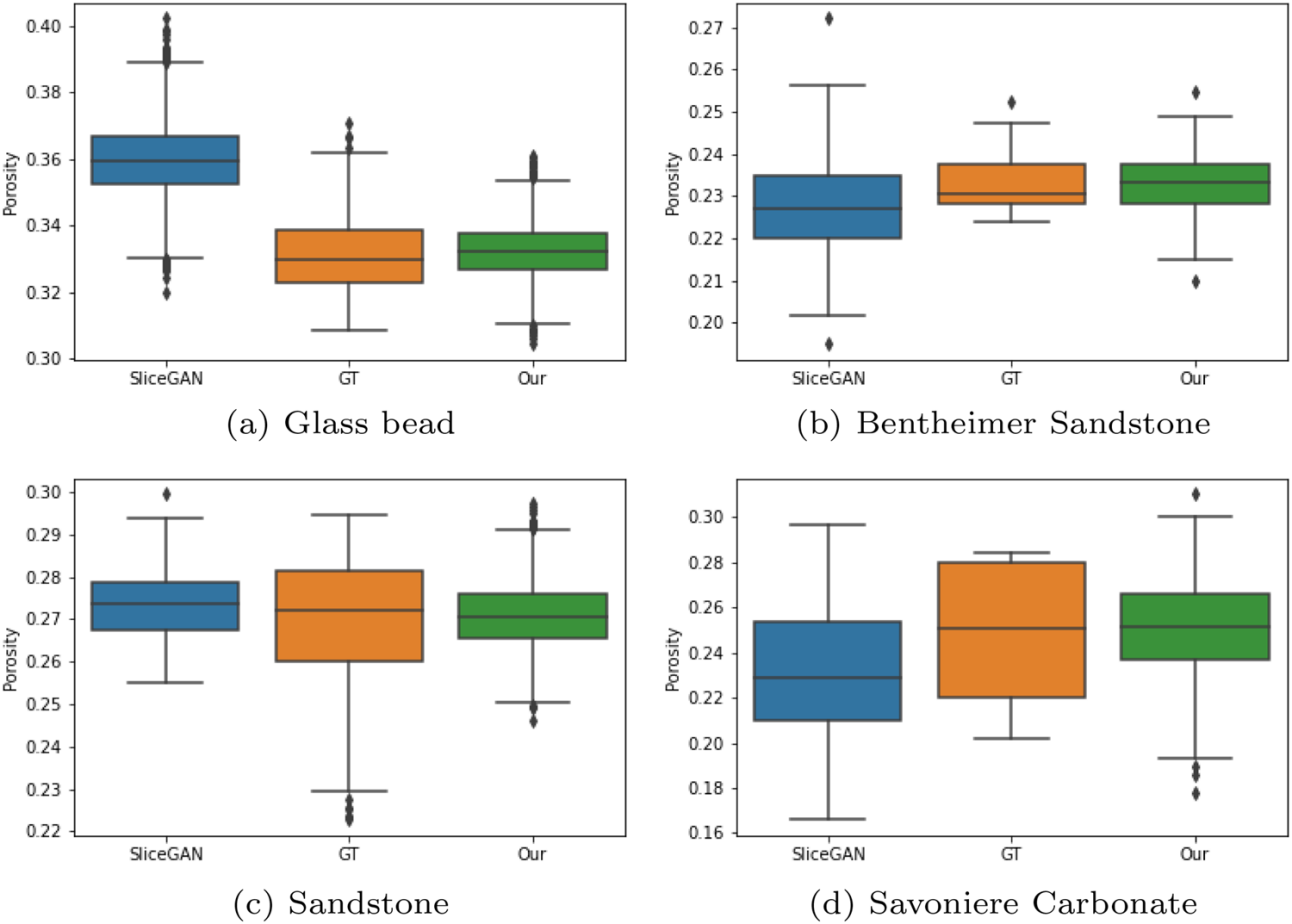
Porosity—these box plots show the comparison of porosity between the ground truth, our model and SliceGAN.

*Porosity* $(\phi )$—Porosity, representing the volume fraction of void spaces, is a fundamental characteristic of porous media. To calculate porosity, we first convert the images into binary format through thresholding. Subsequently, we divide the generated images into overlapping cubes with a side length of 128 voxels. The process of calculating porosity is then applied to these cubes, and the results are visualized using box plots shown in Fig. 5.

**Figure 6:**
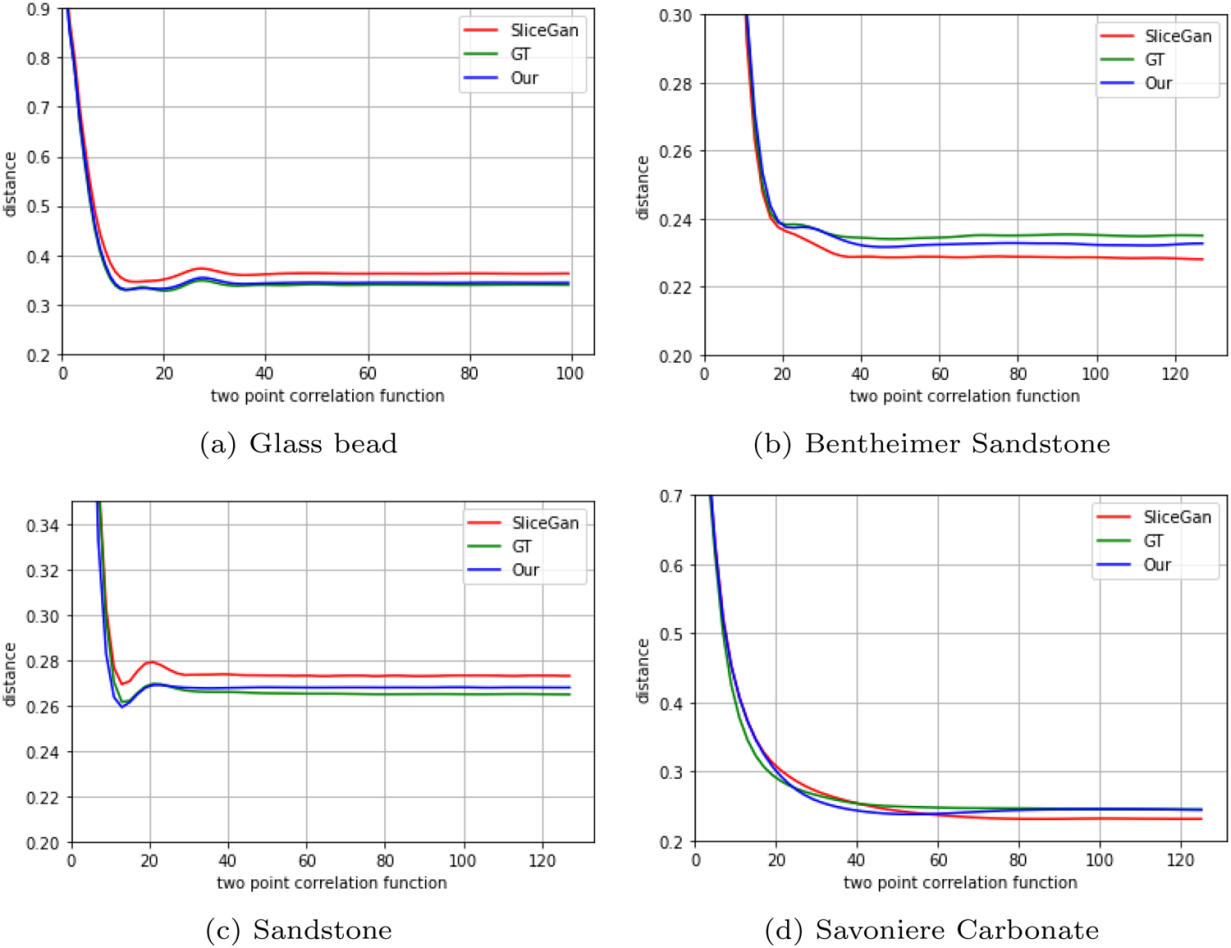
Two-point correlation—these plots depict the two-point correlation function in 3D for the Ground Truth (GT) and the images generated by both SliceGAN and our method. They display the relationship between distance and the probability of a given pixel appearing in a binarized segmented image.

*Two-point correlation function* $(\xi )$—the two-point correlation function is a significant metric in image analysis, utilized to describe the spatial arrangement and connectivity of the porous structure. In this study, we calculated the probability that a pair of points, separated by a certain distance, both reside within the pore space. This statistical measure is sensitive to the image’s degree of homogeneity and isotropy, thus allowing us to capture subtle geometric features of the pore network. The two-point correlation function plots are shown in Fig. 6.

**Figure 7:**
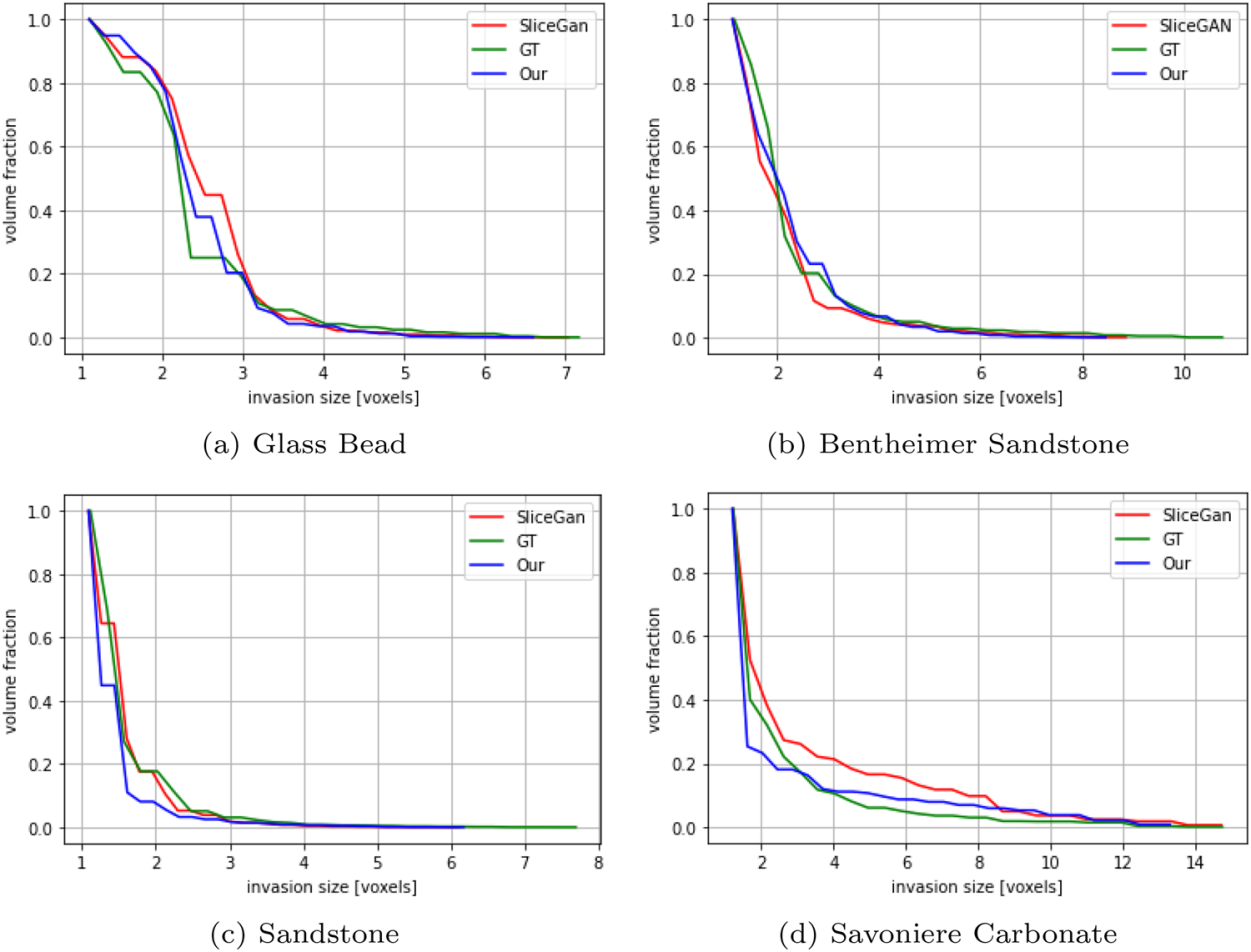
Pore size distribution—these plots shown the comparison of pore size distribution between the generated images and the ground truth. They aid in understanding our model’s effectiveness and SliceGAN’s matching the pore structures to the original 3D micro-CT images.

*Pore size distribution*—pore size distribution is a metric that characterizes the range of pore sizes within a porous material, and it plays a vital role in determining how fluids flow and permeate through the material. Ensuring the accuracy of pore size distribution in our 3D generation from 2D images is important because the sizes and arrangement of pores define the transport properties of the porous medium. Accurate pore size distribution in the generated 3D images is essential for maintaining physical accuracy and predictive usefulness in representing the actual material. We calculated the pore size distribution through a process known as porosimetry, which interprets each voxel in the image as the radius of the largest sphere that would overlap it. For this, we used the Porespy library and the result is shown in Fig. 7.

**Figure 8:**
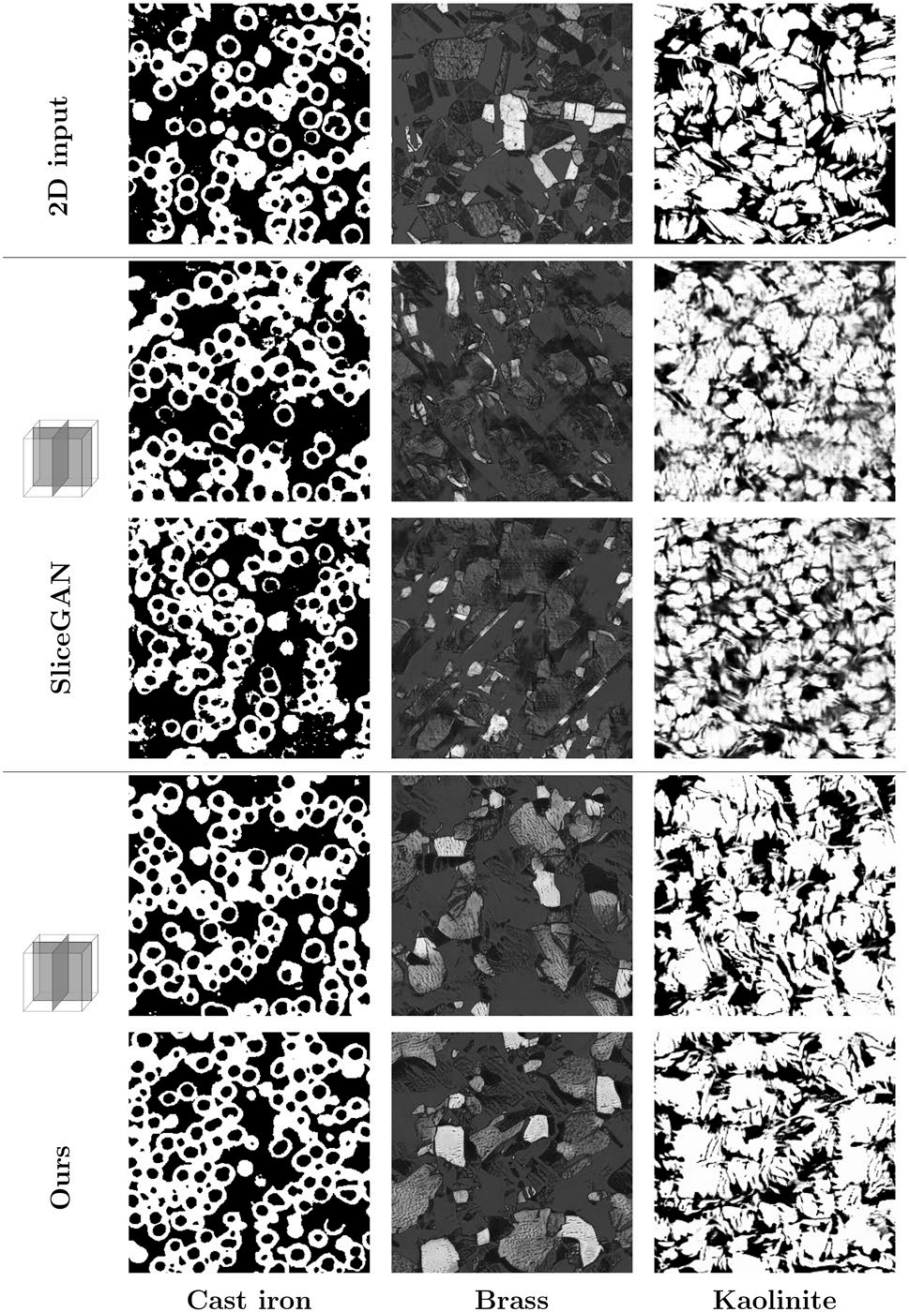
Visual comparison with non-CT data sources: cross-sections of 3D images produced by our method and SliceGAN, presented alongside their respective 2D training images. This figure includes images of cast iron, brass, and kaolinite clay mineral.

### 3D generation experiments from 2D image of a sample

In the final part of our evaluation, we went beyond testing our method’s performance with 3D GT data and tested it in scenarios where only a single 2D image was available, as shown in Fig. 8. We used two metal images (Cast Iron and Brass) from Microlibs^32^, an online database for images generated by *SliceGAN*, as our test data. We trained our model on the downloaded 2D image and subsequently compared our output with both the original training image and the 3D image created by *SliceGAN*, also sourced from the same Microlibs platform. Our study also included an SEM-acquired image of kaolinite clay, recognized for its complex nanostructure that necessitates capturing in 2D. This is due to the high resolution required to capture the sub-microscopic structure of kaolinite, which is beyond the capabilities of current 3D CT scanners.

These cases represent the scenarios where our algorithm may find its most practical use—situations where acquiring 3D images is infeasible, hence the necessity to generate a 3D model from a 2D image.

The comparison in terms of FID score is shown in Table 2.

## Discussion

Our method demonstrates a significant improvement over *SliceGAN*, evident at a visual level, as shown in Figs. 4 and 8. For example, in the glass bead case, our method successfully manages to generate 3D spherical structures from training with 2D circular input, while *SliceGAN* and other machine learning based method failed. In the Sandstone cases, our method demonstrated its capability to handle various resolutions and grain sizes. The Savoniere case, owing to the image’s heterogeneity, presents a challenge in generating a representative 3D image solely from 2D information. Despite this, our method manages to produce a more visually accurate output compared to *SliceGAN* with a significantly lower FID score (Table 1).

Our evaluation of porosity (Fig. 5), the two-point correlation function (Fig. 6), and pore size distribution (Fig. 7), further affirms our method’s efficacy in representing the statistical properties of the 3D GT, even when trained only on five 2D slices. In the final study case where only a single 2D image is available for training and evaluation, we downloaded the 2D image for training and the 3D image created by *SliceGAN* from Microlib. As seen in Fig. 8, our method exhibits versatility by generating high-quality images across different material types. Due to the absence of 3D GT images for these cases, we relied on visual inspection and FID scores for evaluation. Despite the insignificant difference in the visual quality and FID score for the cast iron case, the FID score measured in the Brass case clearly favors our method over *SliceGAN* (see Table 2).

In all study cases included in this work, our method has shown comparable or better performance compared to *SliceGAN*. Additionally, the generated 3D images for materials with simple and homogeneous structures closely match the real images, exhibiting both comparable visual quality and measured properties. However, for more complex and heterogeneous materials, especially those with asymmetrical 3D features, there are areas that indicate potential for improvement. Nevertheless, the results of this study lay a promising foundation for future exploration in the domain of 3D porous media image generation from 2D inputs.

## Conclusion

In this study, we introduced a novel approach to 3D image generation using denoising diffusion probabilistic models (DDPMs) with only a single 2D slice as training data. While DDPMs are not inherently designed for learning from 2D data to represent 3D structures, we introduced a modified reverse diffusion step that effectively denoises a 3D noise vector using a 2D GAN-based discriminator. Our method outperforms state-of-the-art techniques in terms of key physical validation metrics for various types of materials.

Our work marks a significant advancement in the domain of 3D material microstructure generation from 2D inputs. By reducing the dependency on extensive 3D image data and offering a cost-effective, high-resolution alternative to prevailing imaging techniques, our approach paves the way for novel research and practical applications in material characterization and analysis.

## Data Availability

The 2D training datasets presented in this study is publicly available at^32^. The 3D datasets presented in this study available from the corresponding author on reasonable request.
